# Phase 2, Randomized, Open-Label Parallel-Group Study of Two Dosing Regimens of Netarsudil for the Treatment of Corneal Edema Due to Fuchs Corneal Dystrophy

**DOI:** 10.1089/jop.2022.0069

**Published:** 2022-12-02

**Authors:** Richard L. Lindstrom, Amber E. Lewis, Edward J. Holland, John D. Sheppard, John A. Hovanesian, Michelle Senchyna, David A. Hollander

**Affiliations:** ^1^ Minnesota Eye Consultants, Minneapolis, Minnesota, USA.; ^2^ Aerie Pharmaceuticals, Inc., Durham, North Carolina, USA.; ^3^ Cincinnati Eye Institute, Cincinnati, Ohio, USA.; ^4^ Virginia Eye Consultants, Norfolk, Virginia, USA.; ^5^ Harvard Eye Associates, Laguna Hills, California, USA.

**Keywords:** netarsudil, Rho kinase inhibitor, corneal edema, Fuchs corneal dystrophy, central corneal thickness, V-FUCHS questionnaire

## Abstract

**Background:**

This phase 2 study evaluated the therapeutic potential of netarsudil to reduce corneal edema and to improve vision in patients with Fuchs corneal dystrophy (FCD).

**Methods:**

Patients (*N* = 40) with baseline central corneal thickness (CCT) of ≥600 μm and best-corrected visual acuity (BCVA) of 70–20 letters (20/40–20/400 Snellen equivalent) were randomized 1:1 to receive netarsudil once a day (QD) or twice a day (BID) for 8 weeks. Primary endpoint was mean CCT change from baseline at week 4.

**Results:**

Netarsudil QD and BID significantly reduced CCT at week 4 [mean change (standard error of mean), 28.4 (7.99) μm, *P* = 0.0021; and 20.1 (8.75) μm, *P* = 0.0335, respectively]. Five (12.5%) patients achieved complete resolution of corneal edema at week 4. BCVA improved by 3.2 (2.76) letters with QD and 1.5 (2.84) letters with BID, and 10 (25%) patients [5 with QD (*P* = 0.0078) and 5 with BID (*P* = 0.0096)] gained ≥10 letters at week 4. Improvements in CCT and vision were observed at week 2 and persisted at week 8, without significant differences between the 2 doses at any time point. Netarsudil QD significantly improved visual acuity and glare factor scores on the Visual Function and Corneal Health Status (V-FUCHS) questionnaire at weeks 4 and 8 (mean change, −0.4 to −0.3; *P* ≤ 0.0200). Netarsudil was well tolerated. Reticular edema developed in one (2.5%) patient with BID, which resolved with treatment discontinuation.

**Conclusions:**

Netarsudil QD led to significant reductions in corneal edema as well as improvements in vision and patient-reported symptoms of glare and visual impairment in patients with FCD.

Clinical Trial Registration Number: NCT04498169.

## Introduction

Fuchs Corneal Dystrophy (FCD) is a slow, progressive disease that affects the corneal endothelium in both eyes.^1–3^ FCD is characterized by the accumulation of guttae or extracellular collagenous deposits on Descemet's membrane and the dysfunction and gradual loss of corneal endothelial cells.^1–3^ As FCD progresses, endothelial deterioration compromises the ability of the cornea to pump out excess fluid from the stroma, resulting in corneal edema and clouding.^1–3^ Patients with FCD typically present with glare, blurry vision, and a hazy cornea. Decreased visual acuity may be more pronounced in the morning due to increased corneal hydration that occurs from closed eyelids overnight.^3^ FCD is the most common indication for corneal transplantation, accounting for 39% of all corneal transplants performed.^4^

Currently, the only definitive treatment option to restore vision in patients with FCD is surgery.^3^ Endothelial keratoplasty is the gold standard, but it requires using donor corneas.^3^ A newer surgical procedure, called Descemet stripping only (DSO) or descemetorhexis without endothelial keratoplasty (DWEK), is available for select patients and does not require donor corneal tissue.^5^ Many potential complications are associated with surgery, including infection, graft rejection, cataract formation, and secondary ocular hypertension due to long-term use of topical steroids, as well as treatment failure requiring additional surgical interventions.^3^ There remains a large unmet need for a pharmacological treatment to restore the ability of the corneal endothelium to maintain appropriate corneal hydration, which may delay or prevent the need for surgery in patients with visually significant edema due to FCD.^6^

Rho kinase inhibitors have been shown to promote adhesion, enhance proliferation, and inhibit apoptosis of corneal endothelial cells in preclinical models^7–9^ and may have potential benefit for the treatment of corneal endothelial decompensation.^8,10–14^ Netarsudil is a novel Rho kinase inhibitor currently approved in the United States for the reduction of elevated intraocular pressure in patients with open-angle glaucoma or ocular hypertension.^15^ Off-label use of netarsudil has been reported for treating corneal edema associated with FCD. In a randomized, double-masked pilot study of patients with corneal edema secondary to FCD, the use of netarsudil once a day (QD), compared with the use of placebo, led to significant reduction in central corneal thickness (CCT) within 1 month of treatment.^16^ In clinical trials for ocular hypertension and glaucoma, the most common adverse event associated with netarsudil was conjunctival hyperemia. Rare cases of honeycomb or reticular edema have been reported in patients taking netarsudil, but this edema pattern typically resolves with treatment discontinuation.^17–20^

In this article, we report results from a randomized, phase 2 study that demonstrated the therapeutic potential of 2 dosing regimens of netarsudil, QD or twice a day (BID), to reduce corneal edema and improve visual acuity and patient-reported symptoms of visual impairment in patients with reduced vision secondary to FCD.

## Methods

### Study design and oversight

This 8-week, prospective, randomized, open-label, parallel-group, multicenter, phase 2 study was conducted in the United States. Eligible patients were randomly assigned in a 1:1 ratio to netarsudil 0.02% ophthalmic solution QD or BID dosing regimen through the use of an interactive response technology system. Patients self-administered study treatment daily for 8 weeks. In the QD dosing group, patients were instructed to instill one drop of the study artificial tear (REFRESH PLUS^®^ Preservative-Free Lubricant Eye Drops; Allergan) in their study eye every morning (between 06:00 and 09:00 h), and one drop of netarsudil in their study eye every evening (between 19:00 and 22:00 h). In the BID dosing group, patients were instructed to instill one drop of netarsudil in their study eye every morning and evening. Study visits occurred at screening, baseline, and weeks 2, 4, and 8. At the week 4 visit, any patients randomized to netarsudil QD who, in the opinion of the investigator, had not responded adequately to treatment, were permitted to have their netarsudil dosing frequency increased to BID for the remainder of the study.

The protocol was developed by the sponsor, Aerie Pharmaceuticals, and approved by independent ethics committee/institutional review boards at each site. The study was conducted in accordance with Good Clinical Practice guidelines of the International Council for Harmonization and the provisions of the Declaration of Helsinki. All patients provided written informed consent.

### Eligibility criteria

Eligible patients were aged ≥18 years with visually significant central corneal edema due to FCD, in at least one eye, for no longer than 1 year of duration. Key inclusion criteria included a CCT of at least 600 μm at both screening and baseline, as assessed by ultrasound pachymetry, best-corrected visual acuity (BCVA) using the Early Treatment of Diabetic Retinopathy Study (ETDRS) methodology of 70–20 letters (20/40 and 20/400 Snellen equivalent) at screening and baseline, and the presence of central corneal edema deemed by the investigator to be the primary cause of reduced visual acuity. Key exclusion criteria for the study eye included advanced FCD that, in the opinion of the investigator, would likely require surgery within the study period, clinically significant ocular disease (other than FCD) or trauma that could interfere with study interpretation, and history of ocular surgery within 6 months of screening or any prior corneal refractive surgery.

### Study endpoints and assessments

The primary endpoint of the study was mean change from baseline in CCT at week 4. CCT was measured at each study visit by ultrasound pachymetry at approximately the same time of day ±60 min and always before noon (local time). Additional prespecified endpoints included the mean change from baseline in BCVA at weeks 4 and 8, the mean change from baseline in CCT at week 8, the proportion of patients who gained ≥10 (2 lines) and ≥15 letters (3 lines) in BCVA at weeks 2, 4, and 8, and the proportion of patients with complete resolution of corneal edema (defined as a grade 0 for both stromal and epithelial edema) at weeks 4 and 8. In addition, the Visual Function and Corneal Health Status (V-FUCHS) questionnaire was administered at baseline and weeks 4 and 8. The V-FUCHS questionnaire (Supplementary Table S1) is a 15-item, patient-reported, visual disability assessment specifically validated for FCD.^21^ Seven items are related to visual acuity, with “worst” score of 1.77 and “best” score of −0.73, and 8 items are related to glare or diurnal variation, with “worst” score of 1.59 and “best” score of −0.74.^21^

Safety was evaluated at each visit by anterior segment biomicroscopy, ophthalmoscopy, and intraocular pressure. Adverse events were coded using the Medical Dictionary for Regulatory Activities version 23.0.

### Statistical analyses

This study was not powered to detect a pre-stated efficacy signal, but rather was intended to inform the design and power for future studies. With a planned sample size of 20 patients per treatment group, the study was calculated to have 80% power to reject the null hypothesis of “the mean change from baseline CCT in study eyes treated with netarsudil QD or BID at Week 4 = 0” and yield 90% confidence that the true mean change from baseline is within ± standard deviation/2.7 μm of the observed mean change from baseline.

Efficacy and safety were evaluated in all randomized patients who received at least one dose of study medication. The mean changes from baseline in CCT, BCVA, and V-FUCHS scores were summarized using continuous summary statistics; within-group comparisons to baseline were analyzed using one-sample *t*-test. The proportions of patients who gained ≥10 letters (2 lines) or ≥15 letters (3 lines) in BCVA or achieved complete resolution of corneal edema were summarized using discrete summary statistics; within-group comparisons to baseline were analyzed using logistic regression with fixed effects of treatment and corresponding baseline measure. Safety data were summarized descriptively. All data analyses were performed using SAS^®^.

## Results

### Patients

**FIG. 1.**
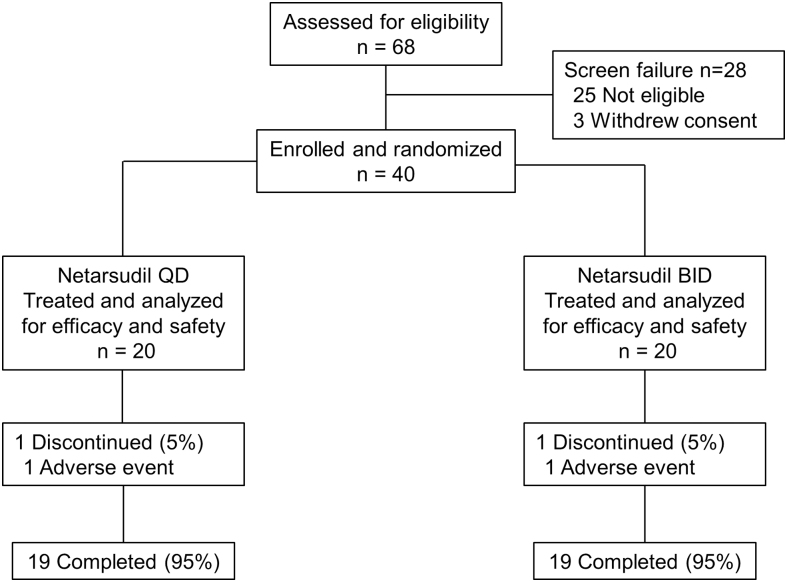
Patient disposition. BID; twice a day; QD, once a day.

A total of 40 patients were enrolled and randomized to receive netarsudil QD (*n* = 20) or BID (*n* = 20) treatment; 19 (95%) patients from each of the original groups completed the study (Fig. 1). A total of 8 patients had netarsudil dosing frequency increased from QD to BID between weeks 4 and 8. At baseline, patients in the netarsudil QD and BID groups had similar visual acuity (mean BCVA, 63.2 letters and 63.3 letters, respectively) and CCT (mean CCT, 669.2 and 660.2 μm, respectively) (Table 1).

**Table 1.** Patient Demographics and Baseline Clinical Characteristics

	Netarsudil QD (*n* = 20)	Netarsudil BID (*n* = 20)
Age (years)		
Mean (SD)	67.9 (10.56)	68.4 (12.32)
Sex, *n* (%)		
Female	9 (45.0)	12 (60.0)
CCT (μm)		
Mean (SD)	669.2 (48.51)	660.2 (49.87)
BCVA (letters)		
Mean (SD)	63.2 (8.19)	63.3 (14.38)

BCVA, best-corrected visual acuity; BID, twice a day; CCT, central corneal thickness; QD, once a day; SD, standard deviation.

### Efficacy

#### Central corneal thickness

**FIG. 2.**
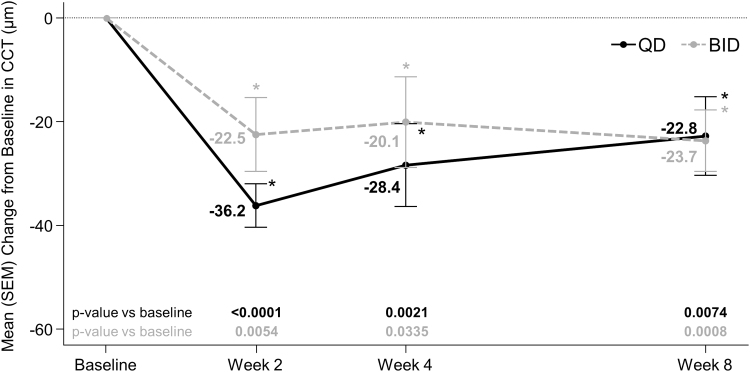
Mean change from baseline CCT in the study eye. *Statistically significant at 5% level. *P* values based on one-sample *t*-test for within-group comparisons to baseline. CCT, central corneal thickness; SEM, standard error of mean.

Netarsudil QD and BID significantly reduced CCT in the study eye from baseline to week 4 (Fig. 2). The mean changes [standard error of mean (SEM)] were −28.4 (7.99) μm in the netarsudil QD group (*P* = 0.0021) and −20.1 (8.75) μm in the netarsudil BID group (*P* = 0.0335) at week 4. Significant reductions from baseline in CTT were observed as early as week 2 and persisted at week 8. There were no significant differences between the 2 dosing groups in changes from baseline in CCT at any time point. Netarsudil QD-treated patients showed greater numerical reductions in CCT at weeks 2 and 4, but not at week 8, compared with netarsudil BID-treated patients. No additional efficacy benefit was seen in the 8 patients who had their dosing frequency increased from QD to BID from week 4 through week 8.

**FIG. 3.**
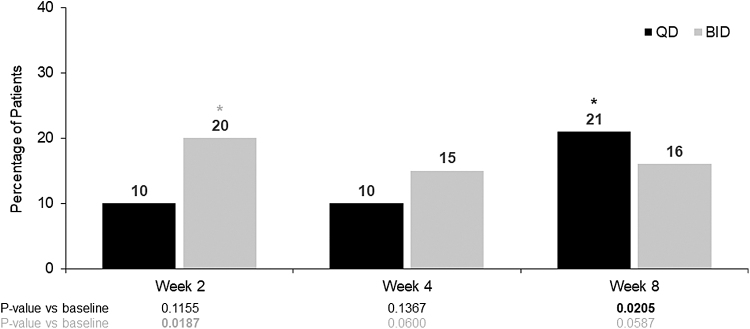
Percentage of patients with complete resolution of corneal edema in the study eye. *Statistically significant at 5% level. Responder analyses: *P* values are based on a logistic regression model fitted with binary outcome variable (Y/N) and baseline mean CCT as a covariate and treatment group as a main effect.

Overall, 5 of 40 (12.5%) patients achieved complete resolution of corneal edema at week 4: 2 of 20 (10%) patients in the netarsudil QD group (*P* = 0.1367) and 3/20 (15%) patients in the netarsudil BID group (*P* = 0.0600). The percentage of patients demonstrating complete resolution of corneal edema was similar between the 2 dosing groups at all evaluated time points (Fig. 3).

#### Visual acuity

**FIG. 4.**
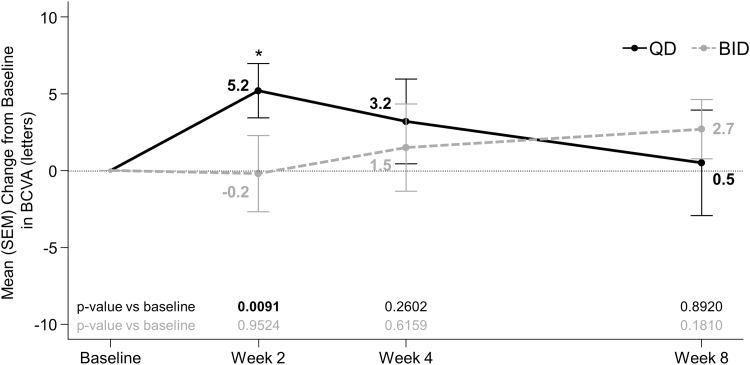
Mean change from baseline BCVA in ETDRS letter score in the study eye. *Statistically significant at 5% level. *P* value based on one-sample *t*-test for within-group comparison to baseline. BCVA, best-corrected visual acuity; ETDRS, Early Treatment of Diabetic Retinopathy Study.

Netarsudil QD and BID improved BCVA by a mean (SEM) of 3.2 (2.76) letters and 1.5 (2.84) letters, respectively, at week 4 (Fig. 4). However, improvements from baseline in BCVA did not achieve statistical significance at any time point except week 2 in the netarsudil QD group. There were no significant differences between the 2 dosing groups in the changes from baseline in BCVA at any time point.

**FIG. 5.**
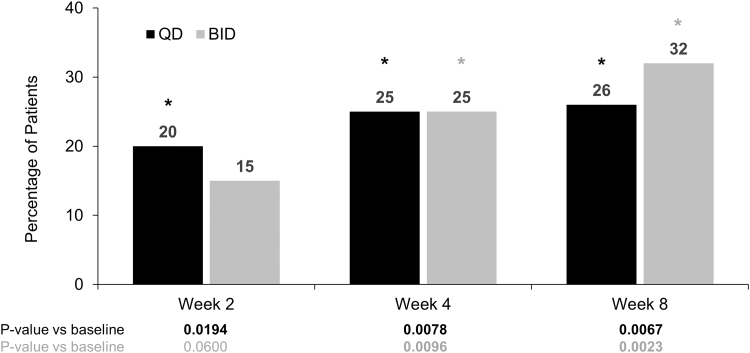
Proportion of study eyes with ≥10-letter (2-line) gain from baseline in BCVA. *Statistically significant at 5% level. Responder analyses: *P* values are based on a logistic regression model fitted with binary outcome variable (Y/N) and baseline BCVA letter score as a covariate and treatment group as a main effect.

Across both dosing groups, 10 of 40 (25%) patients gained ≥10 letters (2 lines), and 6 of 40 (15%) patients gained ≥15 letters (3 lines) in BCVA at week 4 (Fig. 5). A ≥10-letter gain from baseline in BCVA of the study eye was achieved in 5 of 20 (25%) patients in the netarsudil QD group (*P* = 0.0078) and in 5 of 20 (25%) patients in the netarsudil BID group (*P* = 0.0096) at week 4, and sustained in 5 of 19 (26.3%; *P* = 0.0067) and 6 of 19 (31.6%; *P* = 0.0023) patients, respectively, at week 8. Fewer patients gained ≥15 letters (3 lines)—just 3 of 20 (15%) patients in both regimens at week 4, although only 2 patients in the QD group alone maintained this ≥15-letter gain at week 8. The percentage of patients gaining ≥10 or ≥15 letters from baseline in BCVA was also similar between the 2 dosing groups at all time points.

### Patient-reported outcomes

Netarsudil QD and BID treatment improved patient-reported visual disability, as measured by the FCD-specific V-FUCHS questionnaire (Table 2). At baseline, patients in the netarsudil QD group had a numerically worse Visual Acuity Factor Score [mean, 1.07 vs. 0.67 (reported range from worst to best: 1.77 to −0.73^21^)] and Glare Factor and Diurnal Variation Score [mean, 0.72 vs. 0.38 (reported range from worst to best: 1.59 to −0.74^21^)] than those in the netarsudil BID group. Treatment with netarsudil QD led to significant improvements from baseline in V-FUCHS questionnaire Visual Acuity Factor Score at week 4 (mean change, −0.4; *P* = 0.0058) and week 8 (−0.3; *P* = 0.0121) and Glare Factor and Diurnal Variation Score at week 4 (−0.3; *P* = 0.0148) and week 8 (−0.3; *P* = 0.0200). Similar trends were observed with netarsudil BID, but only the reduction in Visual Acuity Factor Score at week 8 reached statistical significance.

**Table 2.** Visual Function and Corneal Health Status Questionnaire

	Netarsudil QD (*n* = 20)	Netarsudil BID (*n* = 20)
Visual Acuity Factor Score, mean ± SEM		
Baseline score	1.07 ± 0.1	0.67 ± 0.15
Change from baseline		
Week 4	−0.39 ± 0.13 (*P* = 0.0058)^*^	−0.14 ± 0.12 (*P* = 0.2252)
Week 8	−0.34 ± 0.12 (*P* = 0.0121)^*^	−0.24 ± 0.13 (*P* = 0.0407)^*^
Glare Factor and Diurnal Variation Score, mean ± SEM		
Baseline score	0.72 ± 0.09	0.38 ± 0.12
Change from baseline		
Week 4	−0.28 ± 0.11 (*P* = 0.0148)^*^	0.04 ± 0.08 (*P* = 0.6300)
Week 8	−0.29 ± 0.11 (*P* = 0.0200)^*^	−0.02 ± 0.10 (*P* = 0.9472)

^*^ Statistically significant at 5% level. *P* values based on one-sample *t*-test for within-group comparisons to baseline.

SEM, standard error of mean.

### Safety

Netarsudil was well tolerated in patients with FCD using both regimens. Over the first 4-week treatment period, 4 of 20 (20%) patients in the QD group and 3 of 20 (15%) patients in the BID group reported adverse events. Six of these 7 patients reported ocular adverse events, 3 in each dosing group. The patient with a non-ocular adverse event in the QD group had a positive COVID-19 test without any additional associated systemic adverse events. A total of 12 ocular adverse events were reported among the 6 patients, including ocular/conjunctival hyperemia (2 events in each dosing group), eyelid erythema (2 in the BID group), corneal staining (2 in the QD group), blurred vision (1 in the QD group), foreign body sensation (1 in the QD group), corneal edema (1 in the BID group), and conjunctival edema (1 in the BID group).

From week 4 to week 8, the period during which patients on the QD regimen were permitted to increase the dosing to BID as per physician instruction, 3 of 11 (27.3%) patients in the QD group and 7 of 27 (25.9%) patients in the BID group reported ocular adverse events. A total of 3 ocular adverse events were reported among the 3 patients in the QD group, including corneal verticillata (2) and ocular/conjunctiva hyperemia (1). A total of 11 ocular adverse events were reported in the 7 patients in the BID group, including corneal verticillata (3), ocular/conjunctival hyperemia (4), corneal edema (1), corneal epithelial microcysts (1), conjunctival edema (1), and eye irritation (1). One of the patients in the BID group also had a non-ocular adverse event of viral gastroenteritis.

Only 2 patients discontinued the study due to adverse events, 1 in the QD group due to blurred vision, ocular hyperemia, and foreign body sensation in eyes, and 1 in the BID group due to the development of corneal epithelial microcysts and worsening corneal edema. In both cases, the adverse events resolved with discontinuation of study medication. The patient with worsening corneal edema in the BID group developed a reticular pattern of edema that resolved 5 weeks after discontinuing netarsudil.

Overall, the majority of adverse events were mild or moderate in severity. Two patients experienced 3 severe adverse events. Blurred vision and ocular hyperemia in one patient in the QD group were considered to be related to the study medication, and both events resolved with treatment discontinuation. The abovementioned viral gastroenteritis in one patient in the BID group (also the only serious adverse event in the study) was not considered related to the study medication.

## Discussion

This phase 2 study investigated the use of netarsudil for the treatment of corneal edema and visual impairment associated with FCD. The results demonstrated that netarsudil was well tolerated and significantly reduced corneal thickness and improved vision in patients with central corneal edema due to FCD. Overall, 25% of patients gained ≥10 letters, with complete resolution of corneal edema observed in 12.5% of patients at week 4. The improvements in corneal edema were observed within 2 weeks of initiating netarsudil therapy, with durable effects in reducing corneal thickening out to week 8. Consistent with objective assessments, patient-reported outcomes were improved at all time points with netarsudil QD dosing.

Netarsudil QD has previously been shown to reduce CCT in a 3-month, placebo-controlled pilot study of patients with FCD.^16^ This phase 2 study confirmed previous results and further demonstrated similar therapeutic effects and safety profiles between netarsudil QD and BID dosing regimens, suggesting that netarsudil QD dosing was acceptable with no added efficacy benefit with more frequent dosing. These results were also consistent with beneficial effects reported from studies of other Rho kinase inhibitors, including ripasudil (available in Japan) and Y-27632 (investigational),^8,10–13^ supporting Rho kinase inhibitors as a class of promising pharmacological treatments for corneal edema due to FCD.

The mechanisms by which netarsudil and other Rho kinase inhibitors may improve corneal edema and vision in patients with FCD are unclear. Based on preclinical studies, Rho kinase inhibitors may promote the progression of corneal endothelial cells in the cell cycle and therefore reactivate cell proliferation and suppress apoptosis. Rho kinase inhibitors may also modulate corneal endothelial cell regeneration by promotion of healthy cell adhesion and migration and restoration of endothelial pump and barrier function.^7–9^ Given the speed of improvement in corneal edema (i.e., resolution within 2 weeks), it is possible that netarsudil may restore the corneal endothelial barrier integrity by inhibiting Rho family small GTPases and reducing leakage across corneal or vascular endothelium and enabling endothelial cell pumps to maintain appropriate corneal hydration,^16,22,23^ among other potential mechanisms of actions.

As a result of the proliferation- and regeneration-promoting properties observed preclinically, the adjunctive use of Rho kinase inhibitors after the surgical removal of Descemet's membrane from the progressive loci in the central region has been reported in patients with FCD and shown to speed the resolution of corneal edema.^14^ Investigational use of netarsudil following DSO/DWEK in patients with FCD has been similarly shown to improve corneal edema, accelerate corneal clearance, and increase corneal endothelial cell counts in a pilot study and several case reports.^24–26^ However, the DSO procedure is irreversible and invasive. Findings from the present study and other studies support the use of Rho kinase inhibitors, including netarsudil, as an alternative to surgery or at least to potentially delay the need for surgery in patients with central corneal edema due to FCD.

The safety of netarsudil has been established in patients with open-angle glaucoma or ocular hypertension.^15^ A similar safety profile was shown in patients with FCD. Netarsudil was well tolerated in both QD and BID regimens, with conjunctival hyperemia, possibly due to vasodilatory effects, as the most common adverse event. Corneal verticillata, which has been previously reported in association with netarsudil and is typically not associated with impact on vision,^15^ most commonly developed after more than 4 weeks of treatment in both treatment regimens. All events of corneal verticillata were mild and either resolved or were reported as resolving at the end of the study without treatment discontinuation or any other actions taken. Honeycomb, or reticular edema, has been reported previously as a rare complication of netarsudil use in anecdotal cases^17–19,27^ and was associated with a history of, or risk factors for, corneal edema.^20^ Reticular edema developed in one patient treated with netarsudil BID in this study and resolved after cessation of treatment, similar to most of the previously reported cases.^17–20^ Reticular edema was not observed with netarsudil QD dosing in this study.

Potential limitations of this study include the lack of control group, relatively small sample size, and short follow-up time. This phase 2 study was conducted without a placebo control group because the previous placebo-controlled study of patients with corneal edema secondary to FCD has shown that netarsudil QD led to significantly greater reduction in CCT than placebo at 1 and 3 months.^16^ More importantly, minimal improvement in corneal edema was observed in the placebo control group in patients with FCD.^16^ This phase 2 study was designed to investigate the optimal dosing regimen for the use of netarsudil in treating patients with visually significant corneal edema due to FCD, and no additional efficacy was observed with BID dosing relative to QD dosing. A larger randomized, controlled study with a longer follow-up is needed to confirm the efficacy of netarsudil for the treatment of corneal edema due to FCD. Netarsudil at current or potentially even lower concentrations may also be investigated as a perioperative topical agent to reduce or prevent corneal edema in cataract and corneal surgery patients.

## Conclusions

The findings of the present study demonstrated that netarsudil QD led to significant reductions in corneal edema as well as improvements in vision acuity and patient-reported symptoms of glare and visual impairment in patients with FCD. These improvements in corneal thickness were typically seen within 2–4 weeks after initiating treatment and then became relatively stable. Further investigations are warranted to explore the therapeutic potential of netarsudil to delay or prevent the need for surgery in patients with corneal edema due to FCD.

## Supplementary Material

Supplemental data
