# Supplementary figures and images for: Phase 2, Randomized, Open-Label Parallel-Group Study of Two Dosing Regimens of Netarsudil for the Treatment of Corneal Edema Due to Fuchs Corneal Dystrophy
Source: J Ocul Pharmacol Ther. 2022 Dec 2;38(10):657–63. doi: 10.1089/jop.2022.0069 (PMC9784611; doi:10.1089/jop.2022.0069)

**Supplemental table 1.**  V-FUCHS Questionnaire21


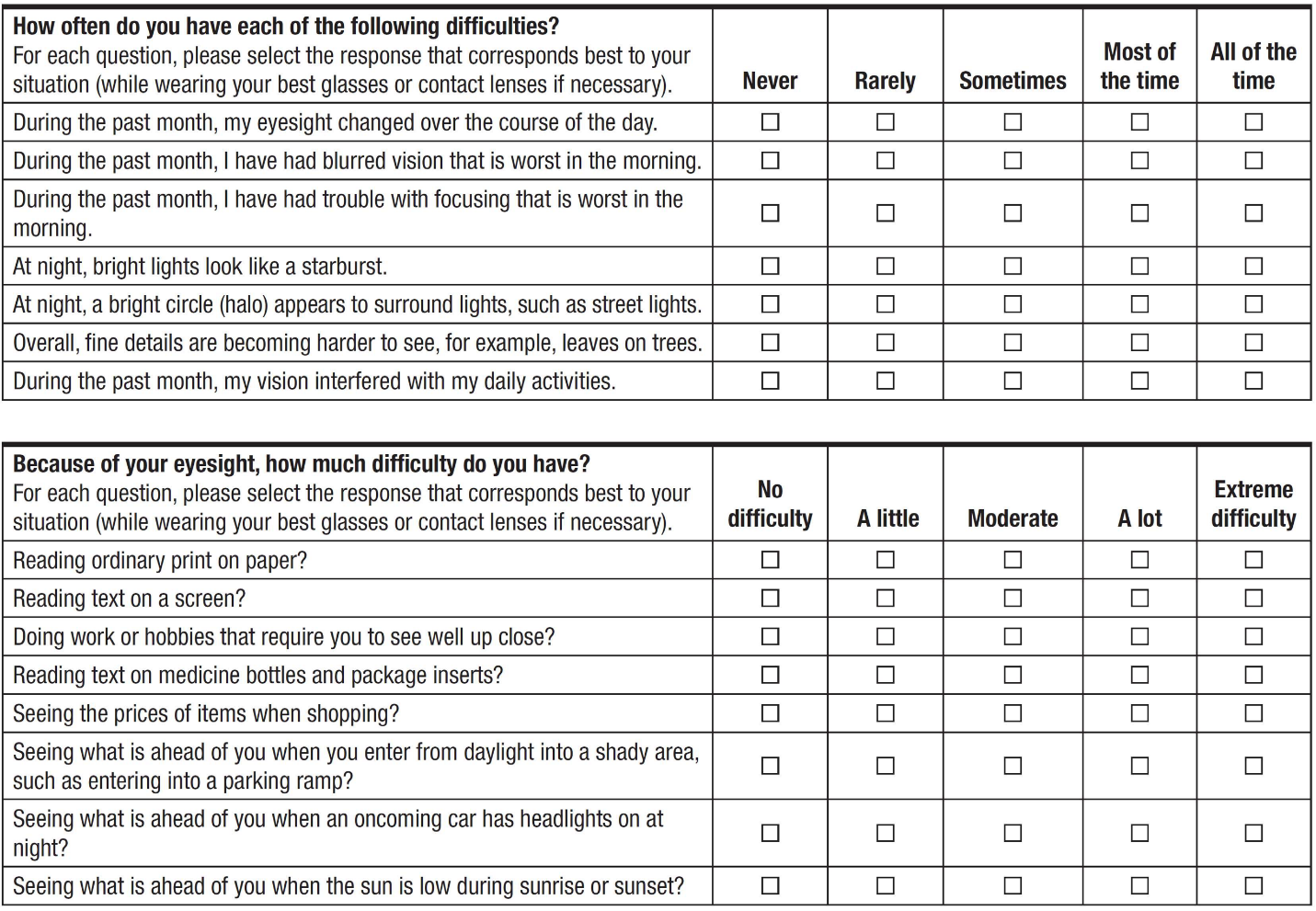

Supplement: Supplemental data [file Supp_TableS1.docx]
